# HIV-1 Tropism Determination Using a Phenotypic Env Recombinant Viral Assay Highlights Overestimation of CXCR4-Usage by Genotypic Prediction Algorithms for CRRF01_AE and CRF02_AG

**DOI:** 10.1371/journal.pone.0060566

**Published:** 2013-05-08

**Authors:** Martin Mulinge, Morgane Lemaire, Jean-Yves Servais, Arkadiusz Rybicki, Daniel Struck, Eveline Santos da Silva, Chris Verhofstede, Yolanda Lie, Carole Seguin-Devaux, Jean-Claude Schmit, Danielle Perez Bercoff

**Affiliations:** 1 Laboratory of Retrovirology, Centre Recherche Public de la Santé, Luxembourg, Luxembourg; 2 Service National des Maladies Infectieuses, Centre Hospitalier de Luxembourg, Luxembourg, Luxembourg; 3 AIDS Reference Laboratory, Ghent University, Ghent, Belgium; 4 Monogram Biosciences Inc., South San Francisco, California, United States of America; The University of Hong Kong, Hong Kong

## Abstract

**Background:**

Human Immunodeficiency virus type-1 (HIV) entry into target cells involves binding of the viral envelope (Env) to CD4 and a coreceptor, mainly CCR5 or CXCR4. The only currently licensed HIV entry inhibitor, maraviroc, targets CCR5, and the presence of CXCX4-using strains must be excluded prior to treatment. Co-receptor usage can be assessed by phenotypic assays or through genotypic prediction. Here we compared the performance of a phenotypic Env-Recombinant Viral Assay (RVA) to the two most widely used genotypic prediction algorithms, Geno2Pheno_[coreceptor]_ and webPSSM.

**Methods:**

Co-receptor tropism of samples from 73 subtype B and 219 non-B infections was measured phenotypically using a luciferase-tagged, NL4-3-based, RVA targeting Env. In parallel, tropism was inferred genotypically from the corresponding V3-loop sequences using Geno2Pheno_[coreceptor]_ (5–20% FPR) and webPSSM-R5X4. For discordant samples, phenotypic outcome was retested using co-receptor antagonists or the validated Trofile® Enhanced-Sensitivity-Tropism-Assay.

**Results:**

The lower detection limit of the RVA was 2.5% and 5% for X4 and R5 minority variants respectively. A phenotype/genotype result was obtained for 210 samples. Overall, concordance of phenotypic results with Geno2Pheno_[coreceptor]_ was 85.2% and concordance with webPSSM was 79.5%. For subtype B, concordance with Geno2pheno_[coreceptor]_ was 94.4% and concordance with webPSSM was 79.6%. High concordance of genotypic tools with phenotypic outcome was seen for subtype C (90% for both tools). Main discordances involved CRF01_AE and CRF02_AG for both algorithms (CRF01_AE: 35.9% discordances with Geno2Pheno_[coreceptor]_ and 28.2% with webPSSM; CRF02_AG: 20.7% for both algorithms). Genotypic prediction overestimated CXCR4-usage for both CRFs. For webPSSM, 40% discordance was observed for subtype A.

**Conclusions:**

Phenotypic assays remain the most accurate for most non-B subtypes and new subtype-specific rules should be developed for non-B subtypes, as research studies more and more draw conclusions from genotypically-inferred tropism, and to avoid unnecessarily precluding patients with limited treatment options from receiving maraviroc or other entry inhibitors.

## Introduction

Entry of the Human Immunodeficiency Virus type 1 (HIV-1) into target cells is a three-step process involving sequential interactions between the viral envelope glycoprotein trimer (Env) with the CD4 receptor and one of two coreceptors, CCR5 or CXCR4 [1]–[7]. Binding to the CD4 receptor induces a series of conformational changes within Env that expose the third hypervariable region (V3-loop), which in turn binds the coreceptor, ultimately leading to the so-called “fusion-active” state required for fusion of the viral and cellular membranes [8]. The V3-loop, which is the main determinant of coreceptor binding, therefore largely accounts for viral tropism [9], [10], and viral strains are classified as R5, when using the CCR5 coreceptor for viral entry, X4 when using CXCR4, and dual-tropic or mixed (R5X4) when using both coreceptors [11]. Other regions of Env, and namely the V1/V2 loops and the constant region C4, have been shown to also participate in viral tropism [12], [13].

R5 strains are generally predominant during the early stages of infection and are thought to be preferentially transmitted by distinct, not yet fully elucidated processes [14], [15]. As infection progresses, viral strains feature increased variability within the infected host, and particularly, Envs acquire broadened coreceptor usage. At late stages of infection, X4 strains become dominant in 50% of patients infected with subtype B strains [16], but subtype-related specificities have been reported [17]–[20]. X4 strains replicate more rapidly than R5 strains *in vitro* and have been associated with increased cytopathicity. *In vivo,* the appearance of X4 strains correlates with a sharp decline of CD4^+^ T cells and the onset of AIDS defining symptoms [21].

With the advent of entry inhibitors targeting CCR5, such as maraviroc, monitoring coreceptor usage has become prerequisite to the prescription of such entry inhibitors, in order to exclude the presence of X4 or R5/X4 variants [22]–[24]. Under maraviroc selective pressure, pre-existing X4 or DM strains can be selected. CCR5 is a cellular target and resistance to maraviroc most often arises through the re-emergence of archived minority X4 strains rather than through a coreceptor usage switch or through the acquisition of mutations that allow gp120 to engage with drug-bound CCR5 [25]–[28]. Viral coreceptor usage can be measured *in vitro* by phenotypic and genotypic assays [29]. Various phenotypic assays based on different techniques are currently available, including the Trofile® Enhanced-Sensitivity-Trofile-Assay (ESTA) (Monogram Biosciences, South San Francisco, CA) [30], the Virco phenotypic test (Virco BVBA, Mechelen, Belgium) and others [30]–[33], which are based on pseudovirions, and assays based on recombinant viruses, among which are the Phenoscript test (VIRalliance, France) [34] and the Toulouse Tropism Test [35]. These assays, their design and performance are summarized in Table 1. The Trofile assay is the most widely used in the clinic. It features a high sensitivity in detecting X4 minority variants [30]. Nonetheless, because phenotypic tests are expensive, time consuming and require specialized laboratories, more interest has been driven toward genotypic testing. Genotypic assays are based on predictions of coreceptor usage from the V3-loop sequence using bioinformatic tools and algorithms. They are currently preferred in Europe due to their accessibility, rapid turn-around and low cost [36], [37]. Many prediction tools are available, with similar specificities and sensitivities despite the fact that they are based on different algorithms involving the 11/25 rule, the number of positively charged AA, the overall net charge of the V3 loop, or combinations thereof. Among them, Geno2Pheno_[coreceptor]_ [38] and webPSSM [39] are the most widely used. European Guidelines for HIV patient management currently recommend the use of Geno2Pheno_[coreceptor]_ with a 10% false positive rate (FPR) cut-off, which has been shown to provide the best balance between specificity and sensitivity for predicting R5 or X4/R5X4 tropism [40]–[43]. The major caveat of such algorithms however lies in the fact that they are based on V3-loop sequences from subtypes B and C mainly, and inadequacies requiring fine-tuning or subtype-specific rules have been reported [19], [44]–[46].

**Table 1:** Characteristics of phenotypic assays developed for determination of HIV-1 coreceptor usage.

Assay		System	Env target	Producer cells	Target cells	readout	Detection limit	reference
	ESTA	Pseudovirions	Full Env	Hek293T	U87.CD4.R5/R4	Luciferase	0.3% X4	[30]
	Virco	Recombinant viral particles	gp120 (NH2-V4)	Hek293T	U87.CD4.R5/R4	eGFP	<10% X4	[32]
Commercial assays	Phenoscript	Recombinant viral particles	V1–V3	Hek293T	U373MG.CD4.R5/X4	β-Galactosidase	5–10% X4	[34]
	PhenXR	Recombinant viral particles	V1–V3	HeLa	SX22-HeLaR5/X4	β-Galactosidase	1% X4	[71]
	Toulouse tropism test (TTT)	Recombinant viral particles	Env Ectodomain	Hek293T	U87.CD4.R5/R4	Luciferase	0.5% X4	[35]
Non-commercial assays		Recombinant viral particles	Full Env	Hek293T	U87.CD4.R5/R4 or GHOST.CD4.R5/X4	Luciferase	1% X4	[72]
		Pseudovirions	Full Env	Hek293T	U87.CD4.R5/R4	Luciferase	1% X4 (high VL)	[33]
							5% X4 (low VL)	
	CRP Env-RVA	Recombinant viral particles	Env ectodomain	Hek293T	U87.CD4.R5/R4	Luciferase	2.5% X4	
							5% R5	

Abbreviations: ESTA: Enhanced Sensitivity Trofile Assay; Env: Envelope; eGFP: enhanced Green Fluorescent protein; X4: CXCX4-using strains; R5:CCR5-using strains.

**Figure 1:**
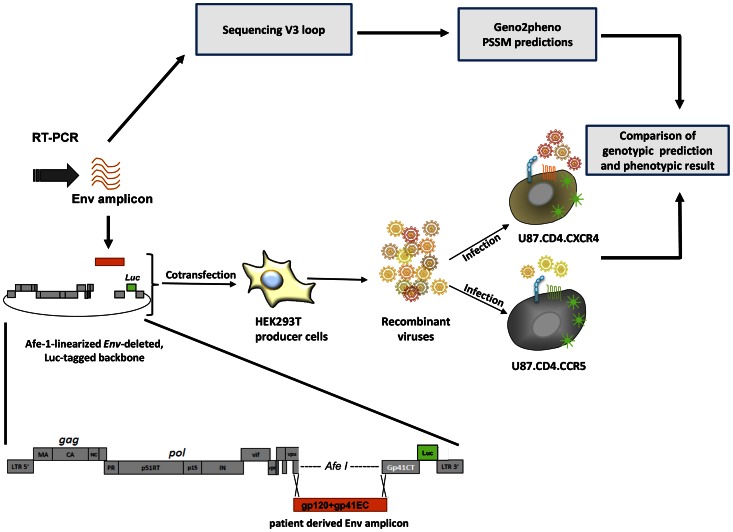
Study design/RVA design. Viral RNA was extracted from patient plasma RT-PCR amplified. Env amplicons spanning the Env ectodomain were further amplified through an inner PCR. Five independent PCRs were pooled to minimize PCR-selection. Recombinant viruses were produced by co-transfecting HEK293T cells with Afe I-linearized, luciferase-tagged, Env-deleted, viral backbone and patient-derived PCR amplicon. Normalized amounts of recombinant viruses were used to infect U87.CD4.CCR5 or U87.CD4.CXCR4 indicator cells. Infection was monitored by quantifying luminescence in the cell lysates. Depending on the outcome of the infection, viruses were classified as either CCR5 tropic, CXCR4 tropic or dual/mixed. The same patient-derived PCR amplicon used for viral production was sequenced and tropism inferred by Geno2Pheno_[coreceptor]_ and webPSSM algorithms. The phenotypic and genotypic results were compared. Abbreviations: Env EC: Env ectodomain; gp41-TM-CT: gp41 Transmembrane+cytoplasmic tail.

This study evaluates the performance of an in-house Env recombinant viral assay (Env-RVA) targeting the full HIV Env ectodomain, in comparison to Geno2Pheno_[coreceptor]_ and webPSSM (Fig. 1). Concordance between the RVA and Geno2Pheno_[coreceptor]_ with a false positive cutoff set at 10% was 85.2% and concordance with webPSSM was 79.6%. Discordant results most often involved non-B subtypes, particularly subtype A1 for webPSSM and CRF01_AE and CRF02_AG for both algorithms. Repeat experiments in the presence of coreceptor antagonists and, when possible, testing the sample using the Trofile ESTA, confirmed phenotypic results of the RVA. Taken together, these results highlight that prediction algorithms are not always accurate for predicting tropism of some subtypes, particularly CRF01_AE and CRF02_AG and underscore the usefulness of maintaining phenotypic testing as well as to adapt algorithms for certain subtypes and recombinant forms.

## Materials and Methods

### Study Population

Plasma samples from 292 patients infected with HIV-1 subtypes B (73), A1 (17), C (21), D (15), F (15), G (55), CRF01_AE (42) and CRF02_AG (54) were included in the study. Left-over plasma obtained from samples for routine clinical tests was used. Ethical approval for use of left-over plasma was obtained from the Comité National d’Ethique pour la Recherche in Luxembourg for HIV assay validation in Luxembourg for HIV assay validation without patient informed consent. The IRB waived the need for written informed consent from the participants for this study. HIV-1 subtypes were determined from HIV-1 PR-RT and Env sequences spanning the V3-loop using COMET (www.comet.retrovirology.lu) and the REGA HIV subtyping tool [47]. CD4 counts ranged from 11 cells/mm^3^ to 1460 cells/mm^3^ (mean: 391 and median: 356 cells/mm^3^). Plasma viral load (VL) (Abbott m2000 RealTime HIV-1 assay) ranged from 466 to 1,350,000 RNA copies/ml for all subtypes, with a mean and median of 14,055 and 71,115 RNA copies/ml respectively. 26/73 subtype B samples with VL <10^3^ RNA copies/mL were also included to assess the RVA’s performance for VL <10^3^ RNA copies/ml.

### Cell Culture

HEK293T cells were obtained from ATCC and were maintained in DMEM medium supplemented with 10% Fetal Bovine Serum, 1% Glutamate, 50 µg/ml Penicillin and 50 µg/ml Streptomycin. U87.CD4.CCR5 and U87.CD4.CXCR4 cells were obtained from the NIH AIDS Reagent Program and were maintained in DMEM containing 10% Fetal Bovine Serum, 1% Glutamate, 300 µg/ml Geneticin and 1 µg/ml Puromycin to maintain CD4 and co-receptor expression respectively. All media supplements and antibiotics were sourced from Invitrogen, Belgium.

### Env Amplification

One ml of plasma or of Env-recombinant virus supernatant was centrifuged at 24,000×g for 1 hour at 4°C and viral RNA was extracted from the pellet using the Qiagen Viral RNA extraction kit (Qiagen, Belgium) according to the manufacturer's instructions. Viral cDNA was synthesized in a one-step RT-PCR reaction using forward primer KVL008 and reverse primer KVL009 [48] in 50 µl mix containing 5 µl viral RNA, 20 µM of each primer, 1 µl SuperScript III One-Step RT-PCR with Platinum *Taq* High Fidelity mix and 8 units RNAseOUT (all from Invitrogen, Merelbeke, Belgium) under the following conditions: initial denaturation at 94°C for 2 mins and 40 amplification cycles (94°C for 15 s, 60°C for 30 s, 68°C for 4 mins) followed by a final 10 mins extension step at 68°C. 2 µl of the amplified cDNA was further amplified using forward primer MM1 FP (5′-GCCTTAGGCATCTCTTATGGCAGGAAGAAG-3′) and reverse primer rec HR1-2_RP (5′-CTCTCTCTfCCACCTTCTTCTTC-3′) [27] in a 50 µl reaction mix containing 2 mM MgSO_4_, 0.2 mM of each dNTP, 20 µM of each primer, 2.5 Units Platinum *Taq* High Fidelity DNA polymerase. The amplification conditions were: initial denaturation step at 95°C for 3 min, 35 cycles of denaturation at 95°C for 30 s, annealing at 48°C for 30 s, extension at 68°C for 3 min, and a final extension step at 68°C for 10 min. Amplification was verified by agarose gel electrophoresis. To avoid PCR selection, five independent amplifications were performed in parallel for each sample and were pooled for recombinant virus production and sequencing.

### Sequencing

To circumvent a potential primer-linked bias, the V3-loop was sequenced directly from the same Env ectodomain amplicon used to produce Env-recombinant viruses (Fig. 1). Sequencing was performed using the BigDye Terminator v3.1 dye on Applied Biosystems 3500 xL DX genetic analyzer (Applied Biosystems Europe BV, Belgium), using sense primers 6951 and 6990 and reverse primers 7336 [49]. For sequencing of viral supernatants, viral RNA was extracted and amplified as described previously and a nested PCR was performed using 2 µl of the Env cDNA, using primers KK1 [50] and DR8 [51] (400 nM each) in a mix containing 5 µl 10× PCR Gold Buffer II, 20 µM dNTPs, 200 µM MgCl2 and 0.5 µl AmpliTaq Gold DNA polymerase (Applied Biosystems), in the following cycling conditions: denaturing for 10 mins at 95°C, followed by 40 amplification cycles (15 sec 95°C, 30 sec 55,5°C, 1 min 72°C) and a final 10 mins extension step at 72°C. For those viral supernatants that could not be sequenced using this method because of inadequate viral content, the viral RNA was amplified and sequenced using primers KK1 and DR8 (400 nM each) in a one-step RT-PCR reaction containing 10 µl viral RNA, 1.5 µl of each primer, 10 µl 5× buffer, 40 µM dNTPs, 0.1 µl RNAse inhibitor and 2 µl Qiagen Taq (Qiagen), as follows: RT: 30 mins at 50°C, denaturation: 15 mins 95°C, 40 cycles of amplification (15 sec 95°C, 30 sec 55.5°C, 1 min 72°C) and a final 5 min extension step at 72°C, followed by an inner PCR using the same conditions as above. V3-loop sequences are available under EMBL Nucleotide Sequence Database with accession numbers: HE972342-HE972511 and JN407569, JN407585, JN407591, JN407601, JN407602, JN407608, JN407609, JN407611, JN407624, JN407629, JN407632, JN407661, JN407676, JN407696, JN407704, JN407706, JN407709, JN407713, JN407726, JN407738, JN407740, JN407745, JN407747, JN407805, JN407808, JN407810, JN407813, JN407814, JN407816, JN407817, JN407836, JN407872, JN407949, JN407971, JN407987, JN407991, JN408004, JN408005, JN408022, JN408023, JN408027, JN408043 and JN408058.

### Genotyping

The V3 nucleotide sequences were submitted to the Geno2Pheno_[coreceptor]_ algorithm (http://coreceptor.bioinf.mpi-inf.mpg.de) setting the FPR set at 5%, 10%, 15% and 20% and to webPSSM (http://indra.mullins.microbiol.washington.edu/PSSM/) using the X4/R5 matrix. The webPSSM subtype C SINSI matrix was used sor subtype C. When mixtures were present in the viral population, all possible combinations were submitted independently to webPSSM and the results were reported as numbers of R5, X4 or R5/X4 clones for comparison with the phenotypic assay.

### Production of Recombinant Viruses

pNL4.3ΔEC.Luc has been described elsewhere [27]. Briefly, pNL4.3ΔEC.Luc is a NL4-3-derived plasmid deleted of the Env ectodomain (6225–8314), containing a AfeI restriction site in the place of Env for linearization and harboring a *firefly luciferase* gene in the place of *nef*. For Env-recombinant viral production, 70% confluent HEK293T cells were co-transfected with Afe I-linearized pNL4.3ΔEC.Luc (Westburg, Netherlands) and patient-derived Env PCR amplicons using Lipofectamine 2000 (Invitrogen, Merelbeke, Belgium) according to manufacturer’s instructions. HIV-1 NL4-3 (X4) and NL-AD8 (R5) were used as positive controls. Cell-free culture supernatants were collected 48 hours post-transfection, clarified by centrifugation and stored at −80°C until use (Fig. 1). Viral production was determined by quantifying p24 capsid protein using a p24 ELISA test (Perkin Elmer, Amsterdam, Netherlands). Virus lacking an envelope produced by transfecting the sole linearized backbone was used to assess background noise.

### Env Recombinant Viral Assay

10^4^ U87.CD4.CCR5 or U87.CD4.CXCR4 cells in 96-well plates were infected with Env-recombinant viruses (200 pg p24, quantified by Perkin Elmer kit) by spinoculation at 1200×g for 2 hours at 25°C [52], followed by incubation for 1 hour at 37°C. Medium was replaced and cells were cultured for a further 48 hours, after which luciferase activity was assayed using the Promega Luciferase assay kit (Promega, Leiden, Netherlands) according to manufacturer’s instructions. Luminescence readout was performed on a Tecan microplate reader (Tecan, Switzerland) over one second exposure. All infections were performed in triplicate. Recombinant viruses were scored as positive for the specific coreceptor if the luciferase signal was at least twice the background.

Where used, 1 µM Maraviroc (CCR5 antagonist) or 1 µM AMD3100 (CXCR4 antagonist) were added to the cells, the plate was centrifuged at 400×g for 10 mins at 25°C and incubated at 37°C for 15 mins prior to addition of the recombinant viruses and spinoculation.

### Trofile ESTA

19 samples (5 subtype B, 2 subtype A1, 2 subtype C, 1 subtype D, 3 CRF01_AE, 6 CRF02_AG) for which phenotypic results differed from the Geno2Pheno_[coreceptor]_-inferred results and for which plasma was available were tested in the Trofile® ESTA.

### Statistical Analyses

In this study, we did not assume the RVA nor the genotypic prediction tools to be the reference test to determine viral tropism, therefore concordance and Cohen kappa values were assessed using Statools (www.stattools.net). Concordance between the phenotypically measured and genotypically predicted tropism was calculated as follows for each subtype or group of subtypes: Concordance = Number of samples with the same tropism by both assays/Total number of samples tested×100. The correlation between tests is usually considered good when kappa>0.6. Tropism measured using the RVA was considered to be concordant with Geno2Pheno_[coreceptor]_ prediction if they both detected pure R5 or pure X4. When dual/mixed strains were detected using the RVA, they were considered to be concordant with an X4 genotypic prediction and discordant with a R5 prediction, as Geno2Pheno only predicts the presence of X4 strains. For comparison with webPSSM, results were considered to be concordant if both assays provided the same result, i.e. R5, X4, or Mixed (D/M). D/M samples detected as a purely X4 or purely R5 sample by the other test were considered to be discordant. Sensitivity and specificity were calculated using GraphPad Prism version 5, setting the phenotypic results as ‘true’.

## Results

### Detection of Minority Variants

**Figure 2:**
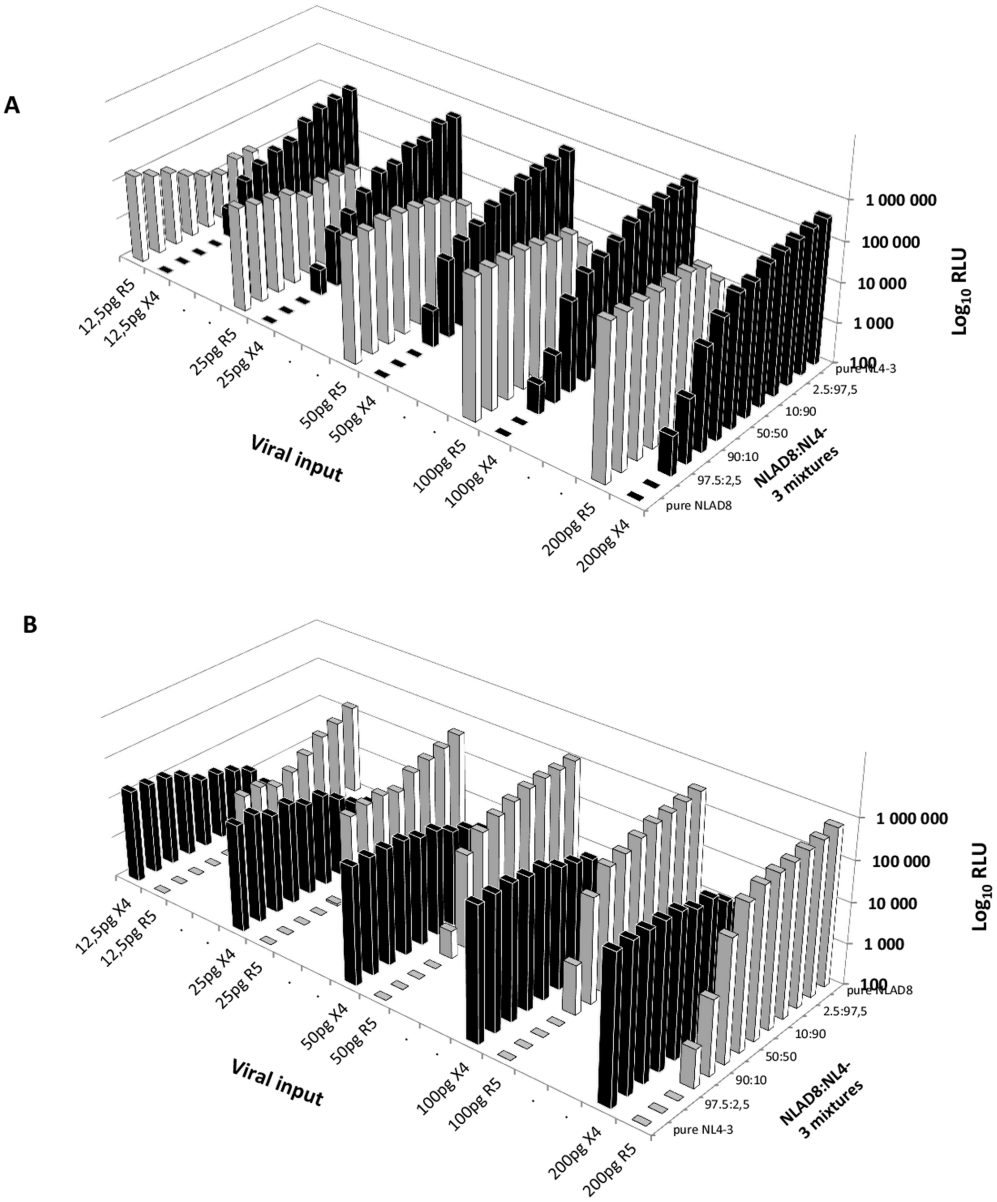
Detection of minority CXCR4 and CCR5 using variants within mixed viral populations. Mixtures containing known proportions of pNLAD8 and pNL4-3 (100∶0, i.e. pure NLAD8, 99∶1, 97.5∶2.5, 95∶5, 90∶10, 80∶20, 50∶50, 20∶80, 10∶90, 5∶95, 2.5∶97.5, 1∶99, 0∶100, i.e. pure NL4-3) were PCR-amplified and used to generate recombinant viruses. U87.CD4.CCR5 and U87.CD4.CXCR4 indicator cells were infected with serial 2-fold dilutions (x-axis) of mixtures (z-axis) to determine the threshold for detection of minority variants. Infection was quantified 48 hours after infection by measuring luciferase activity in cell lysates (y-axis). Black bars report infection of U87.CD4.CXCR4 cells and grey bars report infection of U87.CD4.CCR5 cells. Panels A and B report the same data, oriented to focus on NL4-3 minority variants (A) or on NLAD8 minority variants (B).

To determine the threshold for detecting minority variants, mixtures containing known proportions of pNLAD8 (R5) and of pNL4-3 (X4) were PCR-amplified and used to produce R5/X4 mixed Env recombinant viruses. U87.CD4.CCR5 and U87.CD4.CXCR4 indicator cells were infected with 2-fold serial dilutions of the recombinant NLAD8:NL4-3 mixtures (20 pg to 12.5 pg). As reported in Fig. 2A, 2.5% NL4-3 (X4) minority variants could be detected for NL4-3 with signals higher than 200,000 RLU and 5% NL4-3 variants were detectable with pure NL4-3 signals higher than 50,000 RLU. NLAD8 (R5) minority variants were detected down to 5% at the highest viral input and 10% for control values above 50,000 RLU (Fig. 2B). In this study, experiments were included if positive controls (pure NLAD8 and NL4-3) generated infection signals above 50,000 RLU.

### Production of Patient-derived Env-recombinant Viruses

**Figure 3:**
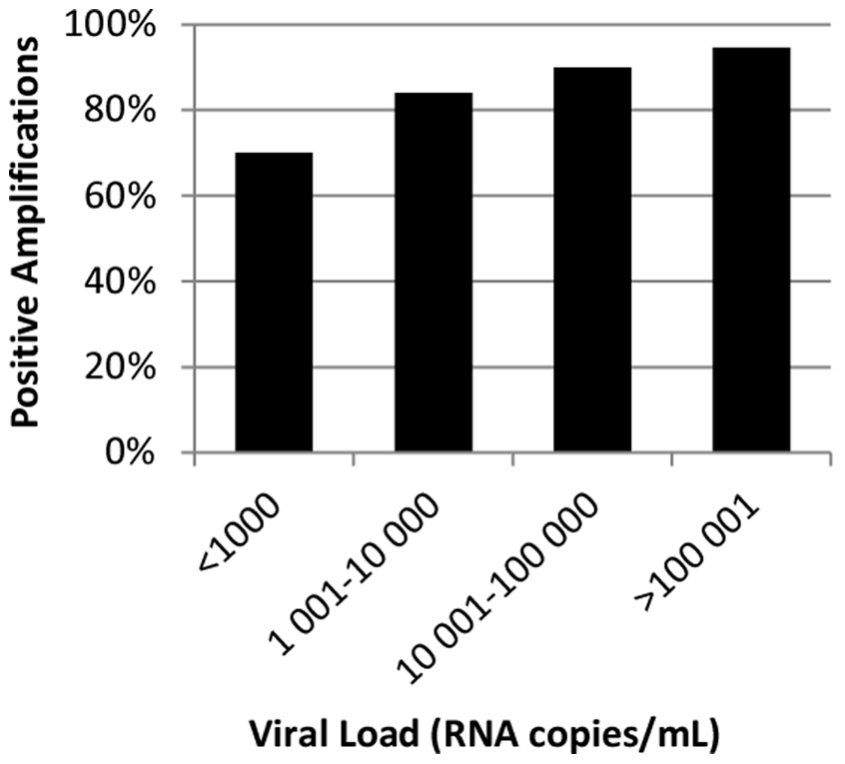
Distribution of PCR amplification success stratified by viral load. The Env ectodomain was amplified from plasma viral RNA by a one-step RT-PCR followed by an inner PCR. Five independent PCR amplifications were pooled to minimize primer-related selection. 292 samples from patients infected with HIV subtypes A1, B, C, D, F, G, CRF01_AE and CRF02_AG were included. Viral load ranged from 466 to 1,350,000 RNA copies/mL.

Overall PCR amplification success of patient-derived Envs was 87% (254/292 samples included) (Table 2). Amplification success was dependent on both subtype and VL. For subtypes A1, B, C, G, and CRF01_AE and CRF02_AG, amplification was achieved in 83.3–100% of cases, while for subtypes D and F, amplification was successful in 66.7% and 46.7% of cases respectively (Table 2). Of note, for some subtypes (D, F) few samples were available (15) inflating the relative weight of failed amplification compared to the overall rate. Both viral load (VL<1,000 RNA copies/mL) and non-B subtype compromised amplification success: the use of subtype-specific primers designed to target the most conserved regions of *env* and based on the most frequent polymorphisms did not improve these figures further (data not shown). When stratified for viral load, amplification succeeded in 94.9% of cases for VL>100,000 RNA copies/mL, 89.7% of cases for VL between 10,001–100,000 RNA copies/mL, 83.8% of cases for VL between 1,000–10,000 RNA copies/mL and decreased to 70% for VL<1,000 RNA copies/mL (Fig. 3). Recombinant viruses could be produced for 231 Envs, as determined by p24 antigen ELISA in the viral supernatant. Of those, 91.3% were infectious and tropism was tested (Table 2).

**Table 2:** Distribution of samples, phenotyping and genotyping, and concordance between phenotypic and genotypic tropism.

Samples		Env PCR		RVA result		V3 loop sequence		Pheno/geno paired results	Concordance of RVA with					Cohen kappa values		
Subtype	N	N	%	N	%	N	%		G2P: 5%	G2P: 10%	G2P: 15%	G2P: 20%	PSSM	G2P: 10%	PSSM	PSSM X4
A1	17	17	100.0%	17	100.0%	15	88.2%	15	93.3%	86.7%	73.3%	73.3%	60.0%	0.4444	0.1743	0.1509
B	73	62	84.9%	62	100.0%	54	87.1%	54	100.0%	94.4%	90.7%	88.9%	79.6%	0.8591	0.3926	0.4752
C	21	21	100.0%	21	100.0%	20	95.2%	20	90.0%	90.0%	85.0%	80.0%	90.0%*	0.6078	0.6190*	0.6078*
D	15	10	66.7%	10	100.0%	7	70.0%	7	85.7%	71.4%	71.4%	71.4%	85.7%	0.3636	0.6111	0.5882
F	15	7	46.7%	7	100.0%	6	85.7%	6	83.3%	83.3%	83.3%	66.7%	83.3%	0.5714	0.5714	0.5714
G	55	52	94.5%	44	84.6%	42	80.8%	40	100.0%	97.5%	95.0%	95.0%	87.5%	0.9180	0.5910	0.8268
CRF01_AE	42	40	95.2%	39	97.5%	40	100.0%	39	79.5%	64.1%	61.5%	61.5%	71.8%	0.3226	0.5153	0.5351
CRF02_AG	54	45	83.3%	32	71.1%	30	66.7%	29	82.8%	79.3%	72.4%	72.4%	79.3%	0.4494	0.0745	0.2077
All non-B	219	192	87.7%	170	88.5%	160	83.3%	156	88.5%	82.1%	77.6%	76.3%	79.5%	0.5446	0.4734	0.5411
**Total**	**292**	**254**	**87.0%**	**232**	**91.3%**	**214**	**84.3%**	**210**	**91.4%**	**85.2%**	**81.0%**	**79.5%**	**79.5%**	**0.6252**	**0.4544**	**0.5240**

Distribution of samples per subtype, successful Env PCR amplification, recombinant virus production and sequencing of the V3-loop, and results (concordance and Cohen kappa values) for genotype/phenotype pairs are reported.

N: number of samples. G2P: Geno2Pheno_(coreceptor)._ The percentage following G2P indicates the FPR cut-off. Distribution of samples per subtype, successful Env PCR amplification, recombinant virus production and sequencing of the V3-loop, and results (concordance and Cohen kappa values) for genotype/phenotype pairs are reported.

* For subtype C, concordance was determined and with webPSSM subtype C SINSI. Concordance with webPSSM X4/R5 was 75% and Cohen kappa values were poor (−0.0989 for full agreement and −0.1365 when detection of X4 strains was considered).

### Concordance of Env-RVA with Genotypic Prediction

Tropism determined phenotypically using the Env-RVA was compared to tropism inferred by the Geno2Pheno_[coreceptor]_ and webPSSM prediction algorithms based on the V3-loop sequence. These two algorithms were chosen among all available genotyping tools because they are the most widely used in the clinic. Results are reported in Table 2. Overall, concordance between the phenotypically measured tropism (RVA) and Geno2pheno_[coreceptor]_ (10% FPR cutoff) was 85.2% and concordance of the RVA with webPSSM was 79.5% (Table 2). The overall Cohen kappa value for comparison with Geno2Pheno was 0.6252, ranging from 0.3226 (CRF01_AE) to 0.9180 (subtype G) (Table 2), reflecting good concordance of the phenotypic measure with genotypic prediction. Overall kappa value for comparison of phenotypically measured tropism with webPSSM was lower (0.4544), ranging from 0.0745 (CRF02_AG) to 0.6190 (subtype C with webPSSM subtype C). If detection of the presence of X4 strains is considered rather than full concordance between the phenotype and the genotype inferred by webPSSM, then concordance between these two assays increased to 83.3% (not shown) and Cohen kappa value reached 0.5240, ranging from 0.1509 (subtype A1) to 0.8262 (subtype G) (Table 2). Among discordant samples, in 14 cases the outcome of both prediction algorithms was identical but disagreed with the phenotypic result, while in the remaining cases, the phenotypically measured tropism disagreed with one algorithm only (Table 3). Decreasing the Geno2Pheno_[coreceptor]_ FPR cutoff to 5% (less sensitive to detect X4) increased concordance to 91.4%, as expected, while augmenting the FPR to 15% and 20% lowered concordance to 81.0% and 79.5% respectively (Table 2).

**Table 3:** Detail of discordant results between RVA, ESTA, Geno2Pheno_(coreceptor)_ and webPSSM.

HIV-1 subtype	RVA result	Trofile result	G2P					webPSSM	Discordance	
			5% cutoff	10% cutoff	15%cutoff	20% cutoff	FPR		G2P	PSSM
A1	R5	R5	R5	X4	X4	X4	6.8	X4	G2P 10%	PSSM
A1	R5	N/A	R5	X4	X4	X4	8.5	D/M	G2P 10%	PSSM
A1	R5	R5	R5	R5	X4	X4	10.5	X4	G2P 15%	PSSM
A1	R5	N/A	R5	R5	R5	R5	42.2	D/M	agree	PSSM
A1	R5	N/A	R5	R5	R5	R5	40.3	X4	agree	PSSM
A1	R5	N/A	R5	R5	R5	R5	22.6	D/M	agree	PSSM
B	R5	R5	R5	X4	X4	X4	6.7	R5	G2P 10%	agree
B	R5	failed	R5	X4	X4	X4	6.8	R5	G2P 10%	agree
B	R5	N/A	R5	R5	X4	X4	13.8	R5	G2P 15%	agree
B	R5	N/A	R5	R5	X4	X4	10.5	R5	G2P 15%	agree
B	R5	N/A	R5	R5	R5	X4	17.6	R5	G2P 20%	agree
B	R5	R5	R5	X4	X4	X4	6.9	R5	G2P 10%	agree
B	D/M	D/M	X4	X4	X4	X4	4.6	R5	agree	PSSM
B	R5	N/A	R5	R5	R5	R5	26.2	D/M	agree	PSSM
B	X4	N/A	X4	X4	X4	X4	0.7	D/M	agree	PSSM partial
B	R5	N/A	R5	R5	R5	R5	72.8	D/M	agree	PSSM
B	X4	N/A	X4	X4	X4	X4	3.7	R5	agree	PSSM
B	X4	N/A	X4	X4	X4	X4	3.8	D/M	agree	PSSM partial
B	X4	N/A	X4	X4	X4	X4	4.7	R5	agree	PSSM
B	X4	N/A	X4	X4	X4	X4	3.8	R5	agree	PSSM
B	X4	N/A	X4	X4	X4	X4	3.7	R5	agree	PSSM
B	X4	N/A	X4	X4	X4	X4	3.8	R5	agree	PSSM
B	X4	N/A	X4	X4	X4	X4	3.8	R5	agree	PSSM
C	D/M	N/A	R5	X4	X4	X4	6.9	R5	G2P 5%	agree
C	R5	R5	R5	X4	X4	X4	9.6	R5	G2P 10%	agree
C	R5	N/A	R5	R5	X4	X4	10.9	X4	G2P 15%	PSSM
C	R5	N/A	R5	R5	R5	X4	17.9	R5	G2P 20%	agree
C	D/M	N/A	R5	R5	R5	R5	28.8	R5	G2P 5%	PSSM
D	R5	R5	X4	X4	X4	X4	4.7	X4	G2P 5%	PSSM
D	R5	R5	R5	X4	X4	X4	6.8	R5	G2P 10%	agree
G	R5	N/A	R5	X4	X4	X4	5	R5	G2P 10%	agree
G	R5	N/A	R5	R5	X4	X4	13.2	R5	G2P 15%	agree
G	D/M	N/A	R5	X4	X4	X4	6.8	R5	G2P 5%	PSSM
G	D/M	N/A	X4	X4	X4	X4	1.3	X4	agree	PSSM partial
G	D/M	N/A	X4	X4	X4	X4	1.7	X4	agree	PSSM partial
G	D/M	N/A	X4	X4	X4	X4	1.1	X4	agree	PSSM partial
G	R5	N/A	R5	R5	R5	R5	26.9	D/M	agree	PSSM
F	D/M	N/A	R5	X4	X4	X4	6.9	D/M	G2P 5%	agree
F	R5	N/A	R5	R5	X4	X4	14.4	R5	G2P 15%	agree
F	R5	N/A	R5	R5	R5	X4	17.5	R5	G2P 20%	agree
F	D/M	N/A	X4	X4	X4	X4	1.7	R5	agree	PSSM
AE	R5	R5	R5	X4	X4	X4	5.3	R5	G2P 10%	agree
AE	R5	N/A	R5	X4	X4	X4	7.9	D/M	G2P 10%	PSSM
AE	R5	N/A	X4	X4	X4	X4	1.8	X4	G2P 5%	PSSM
AE	R5	N/A	X4	X4	X4	X4	2.7	D/M	G2P 5%	PSSM
AE	R5	D/M	X4	X4	X4	X4	1.8	X4	G2P 5%	PSSM
AE	D/M	N/A	X4	X4	X4	X4	1.7	X4	agree	PSSM partial
AE	R5	N/A	R5	X4	X4	X4	9.6	R5	G2P 10%	agree
AE	R5	N/A	R5	X4	X4	X4	5.7	R5	G2P 10%	agree
AE	R5	N/A	R5	X4	X4	X4	5.7	R5	G2P 10%	agree
AE	D/M	N/A	R5	X4	X4	X4	8.7	D/M	G2P 10%	agree
AE	R5	N/A	R5	X4	X4	X4	5	D/M	G2P 10%	PSSM
AE	R5	N/A	R5	R5	X4	X4	10.5	R5	G2P 15%	agree
AE	R5	N/A	R5	R5	X4	X4	10.5	R5	G2P 15%	agree
AE	R5	N/A	R5	R5	X4	X4	12	R5	G2P 15%	agree
AE	R5	N/A	X4	X4	X4	X4	4.1	D/M	G2P 5%	PSSM
AE	D/M	N/A	R5	R5	R5	R5	77.2	R5	G2P 5%	PSSM
AE	R5	N/A	X4	X4	X4	X4	4.7	X4	G2P 5%	PSSM
AE	D/M	N/A	R5	R5	X4	X4	10.5	R5	G2P 5–10%	PSSM
AE	D/M	N/A	X4	X4	X4	X4	1.7	X4	agree	PSSM partial
AG	R5	R5	R5	X4	X4	X4	6.4	R5	G2P 10%	agree
AG	R5	R5	R5	X4	X4	X4	9.6	R5	G2P 10%	agree
AG	R5	N/A	R5	R5	X4	X4	13.8	R5	G2P 15%	agree
AG	R5	R5	R5	R5	X4	X4	10.2	R5	G2P 15%	agree
AG	R5	R5	X4	X4	X4	X4	4.8	R5	G2P 5%	agree
AG	R5	N/A	X4	X4	X4	X4	2.6	R5	G2P 5%	agree
AG	R5	N/A	R5	X4	X4	X4	6.8	R5	G2P 10%	agree
AG	D/M	R5	R5	R5	R5	R5	40.7	R5	G2P 5%	PSSM
AG	D/M	N/A	R5	X4	X4	X4	5.8	R5	G2P 5%	PSSM
AG/G	R5	R5	R5	R5	X4	X4	10.1	R5	G2P 15%	agree
AG	D/M	N/A	X4	X4	X4	X4	1.7	R5	agree	PSSM
AG	D/M	N/A	X4	X4	X4	X4	0.5	X4	agree	PSSM partial
AG	R5	N/A	R5	R5	R5	R5	39.6	D/M	agree	PSSM
AG	D/M	N/A	X4	X4	X4	X4	5.3	R5	agree	PSSM

G2P: Geno2Pheno; FPR: False Positive Rate; D/M: dual mixed N/A = sample was not analyzed; PSSM ‘partial’ refers to samples for which webPSSM agrees with the phenotypic result for the detection of X4 variants, but not on the presence or absence of CCR5-using variants.

For all discordant results (FPR cutoff up to 20% for Geno2Pheno_[coreceptor]_ and webPSSM) for which enough material was available (50/74 samples) the phenotypic assay was repeated using CCR5 and CXCR4 inhibitors (1 µM Maraviroc and 1 µM AMD3100 respectively) (not shown). In all but two cases, the use of coreceptor inhibitors confirmed tropism. In one case, detection of X4 minority variants was close to the limit of detection and was not confirmed in the repeated experiment. In the second case, a strictly R5 strain by phenotypic measure and webPSSM, Maraviroc inhibited infection by 3 logs but did not fully inhibit entry in U87.CD4.CCR5 cells. To rule out the possibility that an intrinsic bias in the recombination step of the RVA would result in selection of some strains and in failure to detect some minority X4 strains, we sequenced the V3-loop of Env-recombinant viral particles used to infect U87 indicator cells. For all re-tested samples (50), the V3-loop sequence of recombinant viral supernatants was identical to the V3-loop of the parental PCR amplicon used to produce the recombinant viruses, and when submitted to Geno2Pheno_[coreceptor]_, a similar or identical FPR was obtained (data not shown), strongly arguing against the hypothesis of a selection due to the recombination process while generating recombinant viruses. Further, for 18 discordant samples, tropism was determined using the Trofile ESTA, which is based on pseudovirions rather than on recombination. Tropism measured using the Trofile ESTA confirmed the RVA results in 16/18 positive tests (Table 3). In one case (CRF01_AE) the RVA failed to detect a minority X4 strain, reflecting the low infectivity of this recombinant, whereas in the second case (CRF02_AG), the RVA detected the presence of low X4 variants which were undetectable using the Trofile ESTA, potentially a consequence of PCR selection due to the sample’s plasma viral load being near the limit validated for the assay.

### Characterization of Discordant Outcomes

**Figure 4:**
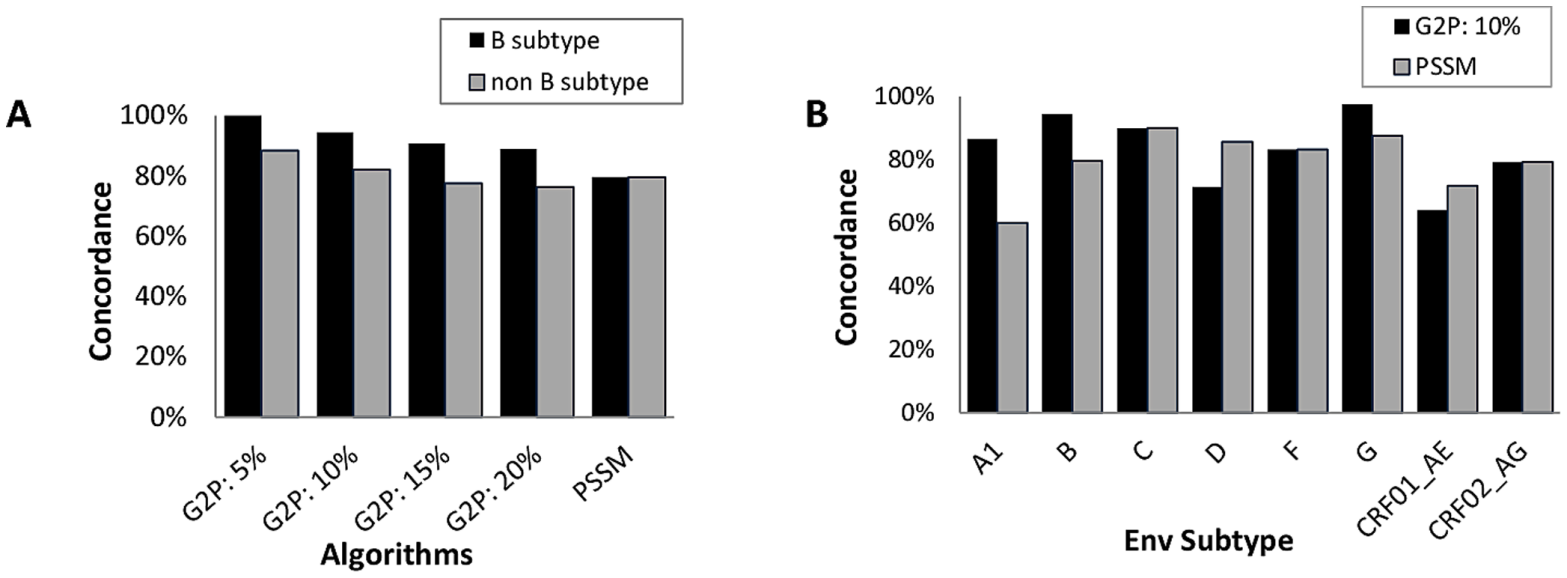
Concordance between tropism measured phenotypically and inferred genotypically using the Geno2pheno_(coreceptor)_ and webPSSM algorithms. (**A**) Concordance for subtype B (black bars) and non-B subtype (grey bars) strains with Geno2Pheno (G2P) at different FPR cutoffs and webPSSM. (**B**) Concordance with Geno2pheno_(coreceptor)_ with a FPR set at 10% (black bars) and webPSSM (grey bars) for different HIV-1 subtypes. The webPSSM X4/R5 matrix was used for all subtypes, except for subtype C, for which the subtype C SI/NSI matrix was used.

Most discordant cases involved non-B subtypes for Geno2Pheno_[coreceptor]_ (10%FPR) (5.6% discordant cases for subtype B, Cohen kappa = 0.8591, versus 17.9% for non-B subtypes, Cohen kappa = 0.5446), but not for webPSSM, for which 20.4% (Cohen kappa = 0.3926) and 20.5% (Cohen kappa = 0.4734) discordant cases were recorded for subtype B and for non-B subtypes respectively (Table 2, Fig. 4). For Geno2Pheno_[coreceptor]_, the highest kappa values were recorded for subtypes G and B, and the lowest value for CRF01_AE. For webPSSM, good kappa values were recorded for subtypes C (using the webPSSM subtype C matrix); using the subtype B X4/R5 matrix, good kappa values were also seen for subtypes D, G, F and CRF01_AE, intermediate for subtypes B, and poor for subtypes A1 and CRF02_AG (Table 2). Of note, kappa values increased to 0.4752 for subtype B when detection of X4 strains was compared rather than absolute concordance (i.e. D/M and D/M, X4 and X4), probably reflecting tuning of the algorithm for the detection of X4 strains.

The sensitivity and specificity of Geno2Pheno_[coreceptor]_ and PSSM with respect to the recombinant viral assay (setting the phenotypic measure as ‘true’) was calculated: overall sensitivity and specificity were 88.9% and 84.2% for Geno2Pheno_[coreceptor]_ (10% FPR cutoff) and 65.2% and 88.4% for webPSSM (considering the ability to detect the presence of X4 variants) (data not shown). For subtype B strains, sensitivity and specificity were 100% and 92.7% respectively for Geno2Pheno_[coreceptor];_ for webPSSM, sensitivity and sensitivity were 46.1% and 95.1% respectively (data not shown). For non-B subtypes, sensitivity was 84.4% for Geno2Pheno_[coreceptor]_ and 72.7% for webPSSM (data not shown). Specificities ranged from 50% to 100% for Geno2Pheno_[coreceptor]_ and from 50 to 100% for webPSSM (data not shown), in line with previous reports [53]–[57].

For subtype B samples, Geno2Pheno_[coreceptor]_ overestimated X4 usage for all 3 discordant samples. In contrast, webPSSM failed to detect CXCR4 usage for 7/11 samples (Table 3). Neither viral load nor the presence of mixtures could account for failure to detect X4 minor strains. For non-B subtypes, Geno2Pheno_[coreceptor]_ predicted CXCR4 usage while the phenotypic assay identified strictly R5 strains in most cases, and particularly for CRF01_AE (12/14 cases) and CRF02_AG (6/7) (Table 3). Such skewing towards overestimating the presence of X4 minority variants for these CRFs was maintained when the FPR cut-off was shifted, although these observations did not reach statistical significance using a Fisher’s exact test (p<0.05). Disagreement of the phenotypically measured tropism with webPSSM was observed chiefly for subtype A1 (40% disagreement) and CRF01_AE (28.2% disagreement). For CRF01_AE, webPSSM predicted CXCR4 usage while the phenotypic RVA reported strictly R5 strains in 7/11 cases, and in 2/11 cases, CXCR4 usage detected phenotypically was missed by webPSSM (Table 3). For subtype A1, CXCR4 usage was overestimated in all cases (6/6 discordant cases) (Table 3).

Overall, both Geno2Pheno_[coreceptor]_ and webPSSM overestimate the presence of X4 viruses for CRF01_AE. A similar trend was also observed for Geno2Pheno_[coreceptor]_ in the case of CRF02_AG and for webPSSM in the case of subtype A1,while webPSSM underestimates the presence of X4 for subtype B. Taken together, these results point to an inadequacy of the genotypic prediction algorithms in correctly inferring tropism for some subtypes CRF01_AE and CRF02_AG, and for subtype A1 in the case of webPSSM.

## Discussion

In this study, the performance of an in-house Env-recombinant viral assay for determining viral tropism was evaluated in comparison to genotypic prediction using 2 widely used algorithms, Geno2Pheno_[coreceptor]_ [38] and webPSSM [39] on a majority of non-B subtypes. Overall, we found good concordance between our phenotypic assay and these algorithms, as reflected by 85.2% concordance of the phenotype with Geno2Pheno_[coreceptor]_ and 79.5% with webPSSM and relative kappa values of 0.6252 and 0.4544 respectively. The highest genotypic/phenotypic concordance was generally found for subtypes G, B and C strains, whereas, despite improved rules, substantial discordances involved non-B subtypes and CRFs. This is in line with previous studies reporting the performance of genotypic tools, mainly webPSSM (X4/R5 and SINSI matrices), Wetcat, Geno2Pheno_[coreceptor]_ or the 11/25 rule, compared to the Phenoscript test or to the Trofile phenotypic assay [53], [58], [59] measuring the sensitivity and specificity of these algorithms to detect the presence of X4 strains. In order to compare our findings with previous reports which evaluated the reliability of different genotypic prediction tools for detecting X4 strains, sensitivity and specificity of these algorithms with respect to the recombinant viral assay were calculated, although this approach presents the intrinsic drawback of setting the phenotypic assay as the standard. We found overall good sensitivities with Geno2Pheno and webPSSM, similar to previous reports on the sensitivity and specificity ranges of different bioinformatics tools for subtype B and some non-B strains [53]–[58]. Of note however, despite similar conclusions, concordance of different genotypic prediction tools with phenotypic assays did not always agree on which algorithm performed best, probably reflecting differences in the panel of viruses and of subtypes included, primer selection, and subsequent comparison with different phenotypic assays targeting the full Env or just the V1–V3 portion. Although the purpose of this study was not to compare the performance of different bioinformatics tools for detecting X4 minority variants but rather to validate our in-house RVA, our findings confirm the high reliability of genotypic prediction tools for detecting the tropism of subtype B strains [53], [54], [56], but also highlight incongruent results for many non-B strains. This has been addressed by webPSSM by developing a specific matrix for subtype C. Concordance of webPSSM with phenotypic results increased from 75% using the webPSSM X4/R5 matrix (based on subtype B) (data not shown) to 90% using the webPSSM subtype C matrix; likewise Cohen kappa values shifted from negative (data not shown) to >0.6 using both algorithms respectively. Therefore, it is important to consider subtype when assessing the presence of X4 strains in the clinical context prior to maraviroc prescription. Our findings strongly argue in favor of using multiple genotypic prediction tools and to consider maintaining phenotypic testing for those subtypes for which coreceptor usage determination using genotypic tests features low concordance with phenotypic measures, and for which prediction algorithms have not been tuned specifically, i.e. non-B, and non-C subtypes.

In this study, genotypic prediction featured the highest discordance with CRF01_AE and CRF02_AG and subtype A1. Of note, Geno2Pheno_[coreceptor]_ and webPSSM did not always predict the same tropism, as previously reported [60]. Geno2Pheno_[coreceptor]_ tended to overestimate the presence of CXCR4 usage (Table 3), while WebPSSM overestimated CXCR4-usage for subtype A1, but not for subtype B or CRF01_AE and CRF02_AG (Table 3). It may be important to keep in mind that when mixtures are present in the V3-loop, all possible combinations are genotyped, and the algorithm provides a tropism prediction for each possible clone. In this scenario, webPSSM would infer tropism for sequences that do not exist in the viral population, eventually leading to an overestimation of dual tropic variants, whereas the phenotypic assays only measures existing strains. Although this phenomenon does not account for mistakenly assigned coreceptor usage, (e.g. strict X4 rather than strict R5), it could explain the improved kappa values recorded for some subtypes when the reliability in detecting the presence of CXCR4-using variants is compared. Such an improvement was particularly marked for subtypes B, G and CRF02_AG (Table 2).

Poor specificity has previously been reported for tropism prediction of subtype D strains by Geno2Pheno_[coreceptor]_ [46] and for CRF02_AG [19], [45], for which specific determinants have been described to improve the algorithm. We therefore subjected our samples to the rules provided by Raymond [45] and those proposed shortly after by Esbjörnsson [19] for CRF02_AG: the Raymond rules, which combine the 11/25 rule and the net charge rule (R/K at position 11 and/or K at position 25, or R at position 25+ net charge ≥+5 or the net charge ≥+6) [45], [61] resulted in 7 discordant cases (20.7%) (3 R5 samples scored as X4 and 3 X4 samples scored as R5) and the Esbjörnsson rule (net charge ≥ +5 and total charged AA ≥8) [19] in 10 discordances (34.5%) (4 missed X4 calls and 6 X4 calls for R5 viruses). Therefore, the Raymond rules slightly improved concordance of genotypic prediction with phenotypic measure for CRF02_AG compared to Geno2Pheno_[coreceptor]_ and webPSSM while the Esbjornsson rules further increased false positive X4 calls on our samples. While this manuscript was under revision, Raymond et al. reported similarly low sensitivity and specificity of Geno2Pheno_[coreceptor]_ (10% FPR cutoff) for CRF01_AE, and proposed a new rule combining the 11/25 rule and disruption of the potential N-glycosylation site PNG (N_6_NT_8_) within the V3-loop [62]. Using a similar approach, we found that in our samples, the presence of a positively charged AA (K or R) at positions 11 or 25 was relatively rare (R/K at position 11 in 6/40 sequences and K at position 25 in one sequence), but reliably translated into CXCR4 usage measured phenotypically. Position 11 hosted a S in 30/40 sequences and a G in 3/40 sequences; position 25 displayed a negatively charged AA (D or E) in 35 sequences. The total number of positively charged AA, total net charge, or total charge, which was generally high (>+5), did not provide further support for sorting CXCR4-using strains in our samples, in agreement with the findings reported by Raymond et al. [62]. In 6/8 phenotypically X4 strains in which positions 11 and 25 were not positively charged, the PNG (N_6_NT_8_) was (or was likely to be, due to mixtures) disrupted and the net charge was ≥+4, as reported by Raymond et al. [62]. The Raymond rules improved concordance of the phenotypic test with genotypic prediction from 61.4% with Geno2Pheno_[coreceptor]_ and 71.8% with webPSSM to 87.2% (34/39) for this CRF. Nonetheless, with these rules (11 K/R and/or 25 K or disrupted PNG+net charge≥+4 [62]) in 2/25 cases, phenotypically R5 samples were scored as X4 and 2/14 phenotypically X4 samples were predicted to be R5, suggesting that other criteria apply to this CRF and larger scale studies combining phenotypic testing to genotypic tuning combining the 11/25 rule to the PNG and to charge will be needed to further improve sensitivity and specificity of prediction tools. We cannot exclude that in our study, the use of bulk sequences may impact the reliability of prediction rules, whereas the rules by Raymond were partially based on clonal samples, where the relative weight of each position is absolute rather than being relative to its proportion within the quasispecies.

Three technical reasons could account for discordant results between genotypic prediction and phenotypically determined tropism: PCR selection, a bias/selection arising from the recombination step of the RVA, inadequately inferred tropism by genotypic tools, consequent to subtype-related specificities not taken into account by the algorithm, either within the V3 loop or in other regions of Env. To minimize a potential impact of PCR selection, 5 independent PCR reactions were pooled and the same amplicon used to produce recombinant viruses was sequenced. Nonetheless, whereas comparison of the Env-RVA and genotypic prediction by Geno2Pheno and webPSSM were based on the use of the same PCR pool, the Trofile ESTA was performed using an independent plasma tube and different PCR primers, eventually translating into PCR selection. Hence, it is not possible to exclude that failure to pick-up X4 minority variants by the RVA (1 CRF01_AE) or by the Trofile ESTA (1 CRF02_AG), reflect PCR selection, particularly in the case of poorly infectious recombinant or pseudotyped particles. Selection during the recombination step was ruled out as the V3-loop sequences from viral supernatants (after the recombination step) were identical to the parental PCR amplicon that served to produce recombinant viruses. When assessed, tropism of CRF01_AE and CRF02_AG strains assessed using the Trofile ESTA matched the phenotype determined by the RVA and disagreed with the Geno2Pheno_[coreceptor]_ prediction in all but two cases (Table 3). The Trofile ESTA was chosen among all available tropism phenotypic tests because it is a high sensitivity and specificity single cycle pseudovirus assay [30]. Lastly, repeat experiments in the presence of CCR5 and CXCR4 inhibitors confirmed the phenotypic results, strongly indicating that the Geno2Pheno_[coreceptor]_ and webPSSM algorithms require more specific improvements for some subtypes, particularly CRF01_AE and CRF02_AG, and that the RVA described here reliably indicates coreceptor usage. It is known that the V3-loop is not the sole Env determinant of HIV-1 co-receptor usage. Sequence changes within the V1, V2 and C4 regions of gp120 [13], [63]–[67], as well as the level of glycosylation [68]–[70] can also profoundly impact coreceptor usage. In this study, it is not possible to rule out that some discordant results arise from the comparison of coreceptor usage predictions based on the V3-loop sequence only to a phenotypic assay taking into account the whole Env ectodomain.

Various commercial and non-commercial phenotypic assays have been developed over the last 10 years to measure tropism. These are based on different approaches to produce recombinant viruses, including homologous recombination, pseudotyping, or a combination of both; they target different parts of Env, ranging from the V1–V3 region only (e.g. Phenoscript [34], PhenXR [71]) to the full Env (e.g. ESTA [30]); the producer and the target cells, as well as the readout (virally-encoded *luciferase* or *GFP* reporter gene (e.g. ESTA [30], TTT [35], the Virco Assay [32], two non-commercial assays utilizing recombinant particles and pseudovirions respectively [33], [72], and the RVA presented here), or target cell line containing a LTR-β-Galactosidase reporter which is activated upon infection (e.g. Phenoscript [34], PhenXR [71]) further distinguish these tests. These are recorded in Table 1. The RVA presented here mostly resembles the TTT assay in the design of the backbone, production of recombinant viral particles through homologous recombination and location of Luciferase reporter in the place of Nef [35]. The TTT showed high performance in terms of Env amplification and production of recombinant viruses, particularly for subtypes that were difficult to amplify using our primers (D and F), likely due to primer location. The TTT group also selected one U87-CD4.CXCR4 cell clone with high expression of CXCR4 to increase the limit of detection of X4 minority variants [35]. In our design, we verified the expression of CXCR4 and only maintained cells in culture for a limited number of passages. To ensure the lower limit of detection of X4 strains was maintained, we tested the performance of the RVA using serial dilutions and systematically discarded experiments where the lower threshold of infection of U87.CD4.CXCR4 cells by NL4-3 did not reach 100,000 RLU (Fig. 2). It would be interesting to compare the performance of different phenotypic assays using a large panel of samples, although this could not be performed here because of insufficient plasma from the sample to allow independent testing and repeat experiments in different laboratories.

Taken together, the findings reported here strongly support the need for further large-scale studies to improve prediction models and/or to appeal to more than one algorithm when non-B subtypes are involved. The use of phenotypic measurements could nevertheless be required in cases where different algorithms point to potential difficulties in inferring the correct tropism. Such a confirmation is important in the clinical set-up as a false prediction of X4 variants may lead to exclusion of patients who could have benefited from prescription of CCR5 inhibitors while false prediction of R5 only variants may lead to selection and reemergence of X4-strains under maraviroc pressure (Baatz et al., 2011; Kuhmann and Hartley, 2008; Pugach et al., 2007; Westby et al., 2007).
