# Correction: HIV-1 Tropism Determination Using a Phenotypic Env Recombinant Viral Assay Highlights Overestimation of CXCR4-Usage by Genotypic Prediction Algorithms for CRRF01_AE and CRF02_AG

**DOI:** 10.1371/annotation/7c0b830d-e75d-4128-ab54-7d5ecfd99d19

**Published:** 2013-07-02

**Authors:** Martin Mulinge, Morgane Lemaire, Jean-Yves Servais, Arkadiusz Rybicki, Daniel Struck, Eveline Santos da Silva, Chris Verhofstede, Yolanda Lie, Carole Seguin-Devaux, Jean-Claude Schmit, Danielle Perez Bercoff

There was an error in the title. The correct title is: HIV-1 Tropism Determination Using a Phenotypic Env Recombinant Viral Assay Highlights Overestimation of CXCR4-Usage by Genotypic Prediction Algorithms for CRF01_AE and CRF02_AG. 

The correct citation is: Mulinge M, Lemaire M, Servais J-Y, Rybicki A, Struck D, et al. (2013) HIV-1 Tropism Determination Using a Phenotypic Env Recombinant Viral Assay Highlights Overestimation of CXCR4-Usage by Genotypic Prediction Algorithms for CRF01_AE and CRF02_AG. PLoS ONE 8(5): e60566. doi:10.1371/journal.pone.0060566. 

